# Particulate matter as a possible risk factor for eosinophilic esophagitis

**DOI:** 10.3389/falgy.2025.1675928

**Published:** 2025-09-18

**Authors:** Natasha Albaneze, Cary C. Cotton, Kristen M. Rappazzo, Charles E. Gaber, Kate Hoffman, Kevin O. Turner, Robert M. Genta, Elizabeth T. Jensen, Evan S. Dellon

**Affiliations:** 1 Department of Epidemiology, Gillings School of Global Public Health, University of North Carolina at Chapel Hill, Chapel Hill, NC, United States; 2 Center for Esophageal Diseases and Swallowing, Division of Gastroenterology and Hepatology, Department of Medicine, University of North Carolina at Chapel Hill, Chapel Hill, NC, United States; 3 Center for Public Health & Environmental Assessment, Office of Research and Development, US Environmental Protection Agency, Research Triangle Park, NC, United States; 4 Department of Pharmacy Systems, Outcomes, and Policy, College of Pharmacy, University of Illinois-Chicago, Chicago, IL, United States; 5 Duke University, Durham, NC, United States; 6 Department of Laboratory Medicine & Pathology, Department of Medicine, University of Minnesota, Minneapolis, MN, United States; 7 Inform Diagnostics, Fulgent, Irving, TX, United States; 8 Center for Gastrointestinal Biology and Disease, Division of Gastroenterology and Hepatology, Department of Medicine, University of North Carolina at Chapel Hill, Chapel Hill, NC, United States; 9 Department of Epidemiology and Prevention, Wake Forest University School of Medicine, Winston-Salem, NC, United States

**Keywords:** eosinophilic esophagitis, EoE, particulate matter, PM_2.5_, PM_10_, environment, exposure

## Abstract

**Background:**

Air pollution, including particulate matter smaller than 10 (PM_10_) and 2.5 (PM_2.5_) µm, increases the risk for heart and lung diseases, including asthma, but has not been extensively studied as a possible etiology in eosinophilic esophagitis (EoE). We aimed to estimate the associations between exposure to PM_2.5_ or PM_10_ and EoE.

**Methods:**

In this case-control study, using a large national pathology database of esophageal biopsies, EoE cases were defined by having biopsies with ≥15 eosinophils per high-powered field in the absence of other histopathologic causes. Controls were all other patients with esophageal biopsies. Patient residential addresses were geocoded and exposure to PM_2.5_ and PM_10_ were estimated using National Emissions Inventory data at the county level for a 5-year period including the biopsy. We estimated the odds ratios (OR) for EoE as a function of PM_2.5_ or PM_10_ exposure in tons emitted per year air using mixed logistic regression models adjusted for individual- and census tract-level characteristics.

**Results:**

Among 12,062 EoE cases and 229,397 non-EoE controls, the unadjusted OR for PM_2.5_ was 1.12 (0.99–1.25) and the adjusted OR was 1.10 (95% CI, 0.99–1.23). The unadjusted OR for PM_10_ was 1.04 (1.00–1.07) and the adjusted odds ratio was 1.02 (95% CI, 0.99–1.06).

**Discussion:**

Exposure to higher levels of PM_25_ and PM_10_ was modestly associated with EoE case status but the association was attenuated by adjusting for potential confounders. The findings suggest any etiologic role for these particulates in EoE would be of small magnitude.

## Introduction

Eosinophilic esophagitis (EoE) is a chronic, immune-mediated condition characterized by symptoms of esophageal dysfunction and infiltration of eosinophils in the esophagus (1). The incidence and prevalence of EoE have been increasing over the past few decades at a rate that outpaces what could likely be explained from increased recognition or increased endoscopy and biopsy rates (2–7). For example, a population-based analysis in Denmark found a nearly 20-fold increase in EoE incidence between 1997 and 2012, with only a 2-fold increase in the esophageal biopsy rate over that same time (5). Although there are known genetic factors that predispose certain individuals to develop EoE, this rapid rise in EoE likely implicates environmental factors as driving the epidemiologic trends (6, 8).

With EoE etiology yet to be fully elucidated, research into environmental risk factors often has stemmed from what is known about other allergic and autoimmune disorders (6). However, there are few studies detailing environmental risk factors in EoE (9–12). Of note, lower population density (13) and worse environmental quality have been shown to be associated with higher EoE prevalence (14), but the reasons for this are unknown. In this context, the role of air quality warrants further investigation, both because current evidence points to a potentially complex relationship between air quality and EoE risk and outcomes (14, 15) and because certain air quality measures, such as particulate matter (PM) concentration, have been shown to be associated with other allergic conditions, such as asthma (16–18). PM comprises a mixture of solid and liquid pollutants found in the air, the concentration of which is routinely measured for two size thresholds (19). PM less than 10 micrometers in aerodynamic diameter (PM_10_) is inhalable and commonly includes dust from industrial and agricultural sites, pollen, and bacterial fragments (19). PM_2.5_ is less than 2.5 micrometers in aerodynamic diameter and often includes emissions from combustion of fuels (19). The sources of PM often differ in rural and urban environments. PM2.5 and PM10 have well-studied adverse impacts on respiratory and cardiovascular morbidity and mortality (20–22), but the gastrointestinal health impacts of PM, and potential differences by size, are less well understood. An umbrella review of meta-analyses of the impacts of air pollution on digestive diseases found some evidence of an association with PM2.5 and colorectal cancer, chronic liver disease, and liver cancer, but no association with esophageal, gastric, or pancreatic cancer (23). The quality of evidence, however, was considered low to moderate, however, and analysis of PM_10_ was lacking (23). The aim of this study was to examine whether living in counties with higher concentrations of PM was associated with increased risk for EoE. Specifically, we investigated this association for PM_2.5_ and PM_10_ emissions and hypothesized that higher emissions would be associated with increased odds of EoE.

## Methods

### Study design and data sources

We conducted a case-control study of patients who underwent upper endoscopy and had esophageal biopsies examined by pathologists at Inform Diagnostics, a pathology laboratory that processes samples from outpatient endoscopy centers across the United States. Biopsies are processed at one of the company's three US-based laboratories (Irving, TX; Phoenix, AZ; Boston, MA) and examined by subspecialty-trained gastrointestinal pathologists using standardized procedures and diagnostic criteria. A detailed explanation of the pathologic examination protocols has been described previously (9, 10, 12–14, 24). This study was deemed exempt from ongoing review by the University of North Carolina Institutional Review Board.

We constructed a database from 701,620 first esophageal biopsies, successfully geocoded 694,626 (99.0%) to United States census tracts, and linked census demographic information to histopathology findings. We geocoded the address data using R (Version 4.1.1, sf package 1.0–16) and linked this to the most recent American Communities Survey (every five years) at the time of biopsy at a census tract-level based on patient residential address. Among the geocoded participants we included 250,401 with biopsies from January 1, 2012 to December 31, 2014 to match the timeframe of exposure data, and limited to 246,950 within the continental United States, including the District of Columbia. We excluded those with missing exposure estimates (2.2%) for any of the five years before case or control definition to yield 241,459 included participants.

### Case and control populations

EoE cases were defined as patients with ≥15 eosinophils per high-power field (eos/hpf; 400× magnification with 22 mm oculars; hpf area of 0.237 mm^2^) on esophageal biopsy, in the absence of other histopathologic causes of eosinophilia (1). Cases were readily identified due to the standardized coding used during pathologic examination, as previously described (9, 10, 12–14, 24). The control group was all patients with esophageal biopsies without EoE. Case definition for incidence was limited by the possibility of having a previous diagnosis of EoE on an outside endoscopy and having uncontrolled EoE on the initial endoscopy in our data.

### Air pollutant exposure metrics

The National Emissions Inventory (NEI) (https://www.epa.gov/air-emissions-inventories/national-emissions-inventory-nei) is a comprehensive summary of air emissions data compiled from multiple sources (primarily state, local, and tribal air pollution control agencies, along with other EPA emissions programs). Major sources for emissions include stationary sources (e.g., electricity generating units, roads), mobile sources (e.g., on-road vehicles, aircraft), fires (e.g., wildfires), and naturally occurring emissions (e.g., vegetation). Emissions are reported in the NEI per source category in tons per year, and NEIs are released on a three-year schedule. For our analysis, we utilized NEIs for 2008, 2011, and 2014 to best correspond to the pathology data years. Emissions were summed across sources to get an estimate of total emissions in tons for each county; we then used linear interpolation to estimate values for intervening years, designating values as missing if two or more of the NEIs reported the county as missing data. Exposure data were averaged over a 5-year lag from case or control occurrence inclusive of the occurrence year. In consideration of possible confounding due to demographic factors and for adjusted modeling approaches, we linked census tract-level at the year of case or control outcome to demographic and economic data, including age, sex, race, ethnicity, income characteristics, and population density from the United States Census or American Community Survey to the exposure and outcome data.

### Statistical analyses

We described, using mean (standard deviation) or median (interquartile range) for continuous variables and number and percent for categorical variables, the distribution of individual- and census tract-level demographic characteristics, and pollutant emissions of the cases, controls, and overall population. We performed tabular analysis of differences between cases and control. We performed mixed effects logistic regression with nested random effects for census tract areas within counties for the unadjusted and adjusted odds ratios (OR) and 95% confidence intervals (CIs). ORs were reported per additional ton emitted per year. To address possible collinearity in census tract characteristics as adjustment variables, the variables were simplified using principal components analysis. The population density was always included in adjusted estimates, as were sex and age, and then principal components were added by stepwise forward selection with a retention threshold of *p* less than 0.2.

### Geographical visualization

For EoE case control status the kernel density using a bivariate normal distribution was estimated to use roughly 100-by-100-mile areas, with the density categorized into deciles. These methods were used to show patterns in case-control status without identifying individual geographical information. For PM_2.5_ and PM_10_ levels in tons emitted per year from the NEI this was visualized as a choropleth plot by county.

## Results

From the registry participants (Table 1), 12,062 EoE cases and 229,397 non-EoE controls were included in analyses. Compared with controls, cases were more commonly male (62.2% vs. 42.4%), were younger (43.8 vs. 56.3 years old) and lived in more economically advantaged neighborhoods ($67,513.43 vs. $62,704.86 median family income). Overall neighborhood differences were small in magnitude between cases and controls (Table 2) with the notable exception of census tract population density. Population density, as previously observed (13), was 31.4% lower among the EoE cases.

**Table 1:** Descriptive statistics of the 241,459 included participants with esophageal biopsies reported, characteristics of their census tract of residence at the time of the biopsy, and the estimated five-year particulate matter exposure characteristics of their home address.

	All registry participants (*N* = 626,929)	Participants in included time window (*N* = 250,401)	Participants also in contiguous 48 states (*N* = 246,950)	Participants also with non-missing exposure (*N* = 241,459)
Individual demographic characteristics:				
Age at biopsy—Mean (SD)	55.86 (16.29)	55.73 (16.41)	55.71 (16.44)	55.70 (16.47)
Male sex assigned at birth—*N* (%)	277,291 (44.27)	108,957 (43.51)	107,313 (43.46)	104,777 (43.39)
Female sex assigned at birth—*N* (%)	349,057 (55.73)	141,444 (56.49)	139,637 (56.54)	136,682 (56.61)
Census tract demographic characteristics:				
Median age—Mean (SD)	40.17 (8.44)	40.26 (8.46)	40.29 (8.48)	40.24 (8.52)
Percent male sex—Mean (SD)	0.49 (0.04)	0.49 (0.04)	0.49 (0.04)	0.49 (0.04)
Percent White race—Mean (SD)	0.78 (0.21)	0.78 (0.21)	0.79 (0.20)	0.78 (0.20)
Percent Black or African American race—Mean (SD)	0.09 (0.15)	0.09 (0.15)	0.09 (0.15)	0.09 (0.16)
Percent American Indian or Alaska Native race—Mean (SD)	0.01 (0.03)	0.01 (0.03)	0.01 (0.02)	0.01 (0.02)
Percent Asian race—Mean (SD)	0.05 (0.09)	0.05 (0.09)	0.05 (0.08)	0.05 (0.08)
Percent Hawaiian or other Pacific Islander race—Mean (SD)	0.00 (0.01)	0.00 (0.01)	0.00 (0.01)	0.00 (0.01)
Percent other race—Mean (SD)	0.04 (0.08)	0.04 (0.07)	0.04 (0.07)	0.04 (0.07)
Percent multiple races—Mean (SD)	0.03 (0.03)	0.03 (0.03)	0.03 (0.02)	0.03 (0.02)
Percent Hispanic or Latino—Mean (SD)	0.16 (0.20)	0.16 (0.21)	0.17 (0.21)	0.17 (0.21)
Census tract economic characteristics:				
Population density—Mean (SD)	2.38 (6.96)	2.51 (7.15)	2.50 (7.18)	2.54 (7.25)
Median move-in year—Mean (SD)	2,002.21 (3.74)	2,002.49 (3.24)	2,002.52 (3.16)	2,002.56 (3.15)*
Median family income—Mean (SD)	64,290.18 (29,238.51)	63,652.70 (29,102.04)	63,421.65 (29,089.81)	62,945.03 (28,832.49)
Percent of households below poverty line—Mean (SD)	0.12 (0.09)	0.12 (0.10)	0.12 (0.10)	0.13 (0.10)
Exposure characteristics:				
Five-year mean PM_10_ at home address in tons/year	17.14 (18.18)	16.52 (15.86)	16.54 (15.96)	16.54 (15.96)
Five-year mean PM_2.5_ at home address in tons/year	4.87 (4.25)	4.75 (3.86)	4.76 (3.87)	4.76 (3.87)

* Missing in 0.9%.

**Table 2:** Descriptive statistics and single-variable *p* values for the case control odds ratio for characteristics of participants’ home address among the 241,459 included participants.

	EoE cases (*N* = 12,062)	Non-EoE controls (*N* = 229,397)	*p* for case-control odds ratio^*^
Individual demographic characteristics:			
Age at biopsy—Mean (SD)	44.78 (16.47)	56.27 (16.27)	<0.01
Male sex assigned at birth—*N* (%)	7,503 (62.20)	97,274 (42.40)	<0.01
Female sex assigned at birth—*N* (%)	4,559 (37.80)	132,123 (57.60)	<0.01
Census tract demographic characteristics:			
Median age—Mean (SD)	39.63 (7.53)	40.27 (8.57)	<0.01
Percent male sex—Mean (SD)	48.99 (3.50)	48.85 (3.75)	0.08
Percent White race—Mean (SD)	80.69 (16.70)	78.22 (20.16)	<0.01
Percent Black or African American race—Mean (SD)	8.04 (13.09)	9.33 (15.72)	<0.01
Percent American Indian or Alaska Native race—Mean (SD)	0.65 (1.43)	0.71 (2.48)	<0.01
Percent Asian race—Mean (SD)	4.57 (7.13)	4.81 (8.45)	0.37
Percent Hawaiian or other Pacific Islander race—Mean (SD)	0.13 (0.58)	0.12 (0.54)	0.87
Percent other race—Mean (SD)	3.18 (5.49)	4.10 (7.31)	<0.01
Percent multiple races—Mean (SD)	2.72 (2.36)	2.71 (2.43)	0.77
Percent Hispanic or Latino—Mean (SD)	14.27 (17.80)	16.83 (21.04)	<0.01
Census tract economic characteristics:			
Population density—Mean (SD)	1.77 (4.90)	2.58 (7.35)	<0.01
Median move-in year—Mean (SD)	2,002.74 (3.10)	2,002.55 (3.15)	<0.01
Median family income in contemporary US dollars—Mean (SD)	67,513.43 (29,345.90)	62,704.86 (28,785.26)	<0.01
Percent of households below poverty line—Mean (SD)	11.02 (8.57)	12.60 (9.63)	<0.01
Exposure characteristics:			
Five-year mean PM_10_ at home address in tons/year	17.76 (16.79)	16.48 (15.92)	0.03
Five-year mean PM_2.5_ at home address in tons/year	4.99 (4.09)	4.75 (3.86)	0.08

US, United States. ^*^Wald test for logistic regression model term estimated with iteratively reweighted least squares.

**Figure 1:**
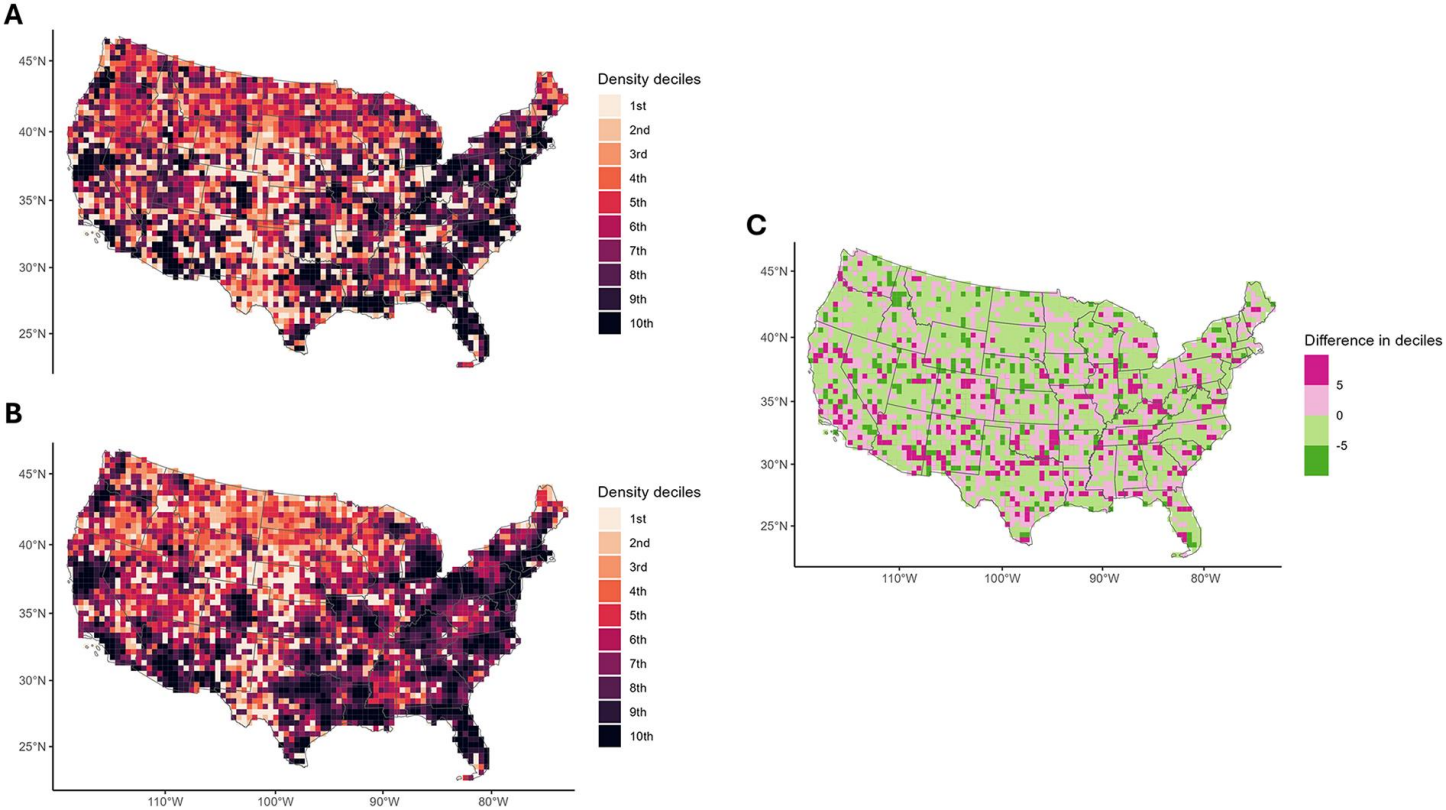
**(A)** Choropleth map of the estimated density of upper endoscopy biopsies with eosinophilic esophagitis (cases) by Gaussian kernel density estimate with approximately 100-by-100-mile quantiles. **(B)** Choropleth map of the estimated density of upper endoscopy biopsies without eosinophilic esophagitis (controls) by Gaussian kernel density estimate with approximately 100-by-100-mile quantiles. **(C)** Choropleth map of the difference in quintile of estimated density between upper endoscopy biopsies with and without eosinophilic esophagitis by Gaussian kernel density estimate with approximately 100-by-100-mile quantiles.

**Figure 2:**
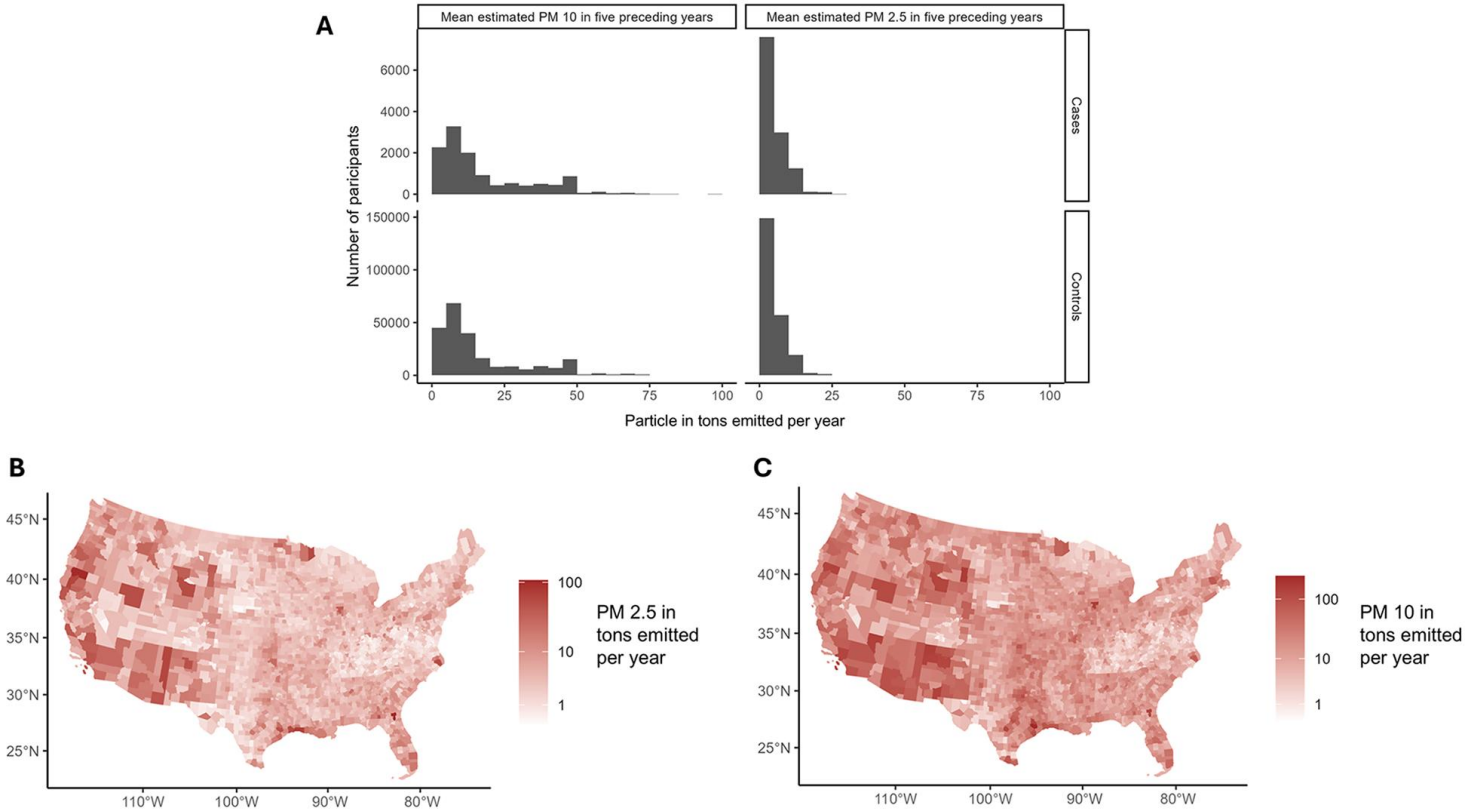
**(A)** Histogram of the primary exposures as the mean of estimated PM_10_ and PM_2.5_ levels in tons emitted per year averaging values in the in the same county for the biopsy year and the four previous years. **(B)** Choropleth map of the estimated 2010 PM_2.5_ estimates by year and contiguous United States county in tons emitted per year. **(C)** Choropleth map of the estimated 2010 PM_10_ estimates by year and contiguous United States county in tons emitted per year.

The estimated geographic distributions of the EoE cases and controls showed a moderate predisposition of cases to less dense locations (Figure 1A–C) The overall distribution of exposure to the size-classes of particulate matter of interest was low, with most participants exposed to less than EPA recommended limits of both PM_2.5_ and PM_10_ (Figure 2A) The 2010 geographic distributions of the primary exposures, PM_2.5_ and PM_10_ levels by county, are graphically represented in Figures 2B,C, where notable heterogeneity by county is observed. Much more subtle changes over the course of the seven years of exposure history for the cohort are shown in Supplementary Figures S1, S2.

In analysis only adjusted with random effects for clustering within counties and census tracts, case status had a small positive association with case vs. control status for PM_2.5_ (OR, 1.12, 95% CI: 0.99–1.25, and a smaller but more precise association for PM_10_ (OR, 1.04, 95% CI: 1.00–1.07) when assessing odds ratio per ton additional estimated particulate pollution per year by county among the 239,361 without any missing covariates. However, after adjusting for age, sex, census tract population density, and the one principal component of other census-tract demographic characteristics retained based on our selection threshold, both the associations between case vs. control status for PM_2.5_ exposure (aOR 1.10, 95% CI: 0.99–1.23), and PM_10_ exposure (aOR 1.02 95% CI: 0.99–1.06) were moderated. Retention of the first two principle components only minimally affected the OR estimates.

## Discussion

With investigations into the evolving epidemiology of EoE suggesting an environmental role in disease development, studies of specific environmental risk factors are needed to better understand EoE pathogenesis (6). In this study of the association between exposure to PM and EoE, we found that exposure to higher levels of both PM_10_ and PM_2.5_ was associated with EoE case status, but this association was of modest magnitude and was attenuated with adjustment. The findings suggest any etiologic role for these particulates in EoE is of small magnitude and does not explain the sharp increase in EoE incidence seen in the past several decades. However, if only certain components of PM contribute to EoE development, aggregation would dilute potentially stronger associations. Thus, the modest association seen in our study should not preclude future investigation of the potential role of air pollution in EoE etiology but does suggest there are other environmental sources that likely have played a larger role in the population-level increase in EoE.

Prior research using the same national pathology database that found EoE to be inversely associated with the air domain of the EPA's Environmental Quality Index (14), of which PM_2.5_ and PM_10_ are components (25). Regarding PM_2.5_, there is evidence that EoE is inversely associated with population density (13). Given that PM_2.5_ concentrations are generally lower in rural/low population-density compared to urban/high population-density areas (26, 27), the known rural predisposition of EoE does align with that as a possible cause. However, adjustment for this made only a modest difference, potentially due to heterogeneity in PM_2.5_ concentration across rural areas, due to the relevance of anthropogenic and natural pollution sources and exacerbating or mitigating factors other than population density (27–29). We are aware of one additional study examining PM_2.5_ and EoE, albeit with a focus on EoE symptoms as opposed to prevalence (15). This case-crossover study of patients in a single state by May Maestas and colleagues found that exposure to elevated PM_2.5_ concentrations was associated with increased odds of emergency department visits for EoE symptoms, such as chest pain, dysphagia, and food impaction, though the possibility for confounding for cardiovascular or asthma presentations remained as well (15).

The study of the association between PM_10_ and EoE or risk factors for EoE has been limited. PM_10_ is thought to make up a larger proportion of PM in rural than urban areas, in general (30), which could help explain the positive association we found between PM_10_ and EoE, a condition with higher prevalence in areas with lower population density (13). However, more research is needed to better understand what feature of PM_10_, such as size/mass or a specific component, contributes to its positive association with EoE, and where, geographically, it may be more prevalent due to natural or anthropogenic sources. These findings suggest that future studies should continue to examine specific sources of air pollution or sizes of PM, as opposed to aggregating results across air pollution types.

Given the pro-inflammatory response elicited by exposure to air pollution, including PM, and the link between air pollution and asthma (31, 32), we had hypothesized the positive association between PM_10_ and EoE seen in our study, but did not necessarily expect a less prominent association between PM_2.5_ and EoE. Data on air pollution's effects on eosinophils, particularly in the esophagus, are scarce, but some data indicate exposure to pollution can be associated with eosinophilic inflammation and trafficking of eosinophils from the blood to the respiratory tract (31). One potential explanation for the variation in esophageal eosinophilic inflammation seen in our study in response to PM_2.5_ vs. PM_10_ exposure could be that PM_10_ generally is deposited in the upper respiratory tract, while PM_2.5_ generally is able to reach lower within the lungs (30, 33–35). Clearance of PM can vary by particle size and location, among other factors, with larger particles more rapidly cleared via mucociliary clearance (MCC) to the throat, compared with smaller particles that often take longer to clear via MCC or, if they reach the alveoli, can be cleared via other mechanisms that may not lead to esophageal PM exposure (33, 36, 37). While the mechanism and timeliness of clearance of PM is complex and influenced by additional factors such as particle density and solubility, as well as PM-induced damage to the airway (34–36, 38), it is possible that a greater proportion of these larger PM_10_ particles could contact the esophagus via swallowing of particles deposited or cleared into the oral cavity. Thus, the degree of immunologic response in the esophagus may differ for PM_10_ vs. PM_2.5_, but further studies are needed to understand the degree to which the esophagus is exposed to PM, including specific components of PM, as well as mechanisms of recruitment of eosinophils to different tissues in response to PM exposure.

There are limitations to our study. Given that there is typically an extended period between EoE symptom onset and diagnosis and wide inter-patient variation in the length of time (39), attempting to evaluate a shorter-term exposure period based on symptom onset would likely result in exposure misclassification. Therefore, our results should be interpreted in the context of cumulative, elevated PM exposure over an extended period. Our use of a patient's address at the time of their biopsy to estimate PM exposure would not account for patients moving across census tracts during the five years before their biopsy or time spent in other census tracts, such as for work, which could result in misclassification of PM exposure levels. Additionally, we use ambient metrics for PM exposure as a proxy for individual-level exposures which does not account for individual-level behaviors, such as time spent outdoors, smoking or living with a smoker, and use of air filtration devices, that influence individual PM exposure, which is another potential source of exposure misclassification. The misclassification may be dependent on relevant individual-level measured and unmeasured covariates, such as age and socioeconomic status, with a lack of available data preventing us from assessing the potential impact of this source of bias. Although we adjusted for selected individual- and census tract-level characteristics, residual confounding is possible, particularly from unmeasured covariates, such as individual socioeconomic status and mobility, respiratory comorbidities, etc. Furthermore, we cannot be certain whether EoE cases are incident or prevalent, which is a limitation of our pathology database that does not allow us to establish the temporality of PM exposure and EoE development. Based on these limitations, our data are not sufficient to establish causality. Our study has several strengths as well, including our use of a large database which includes esophageal biopsies from across the country. Our ability to select controls from this population of patients with esophageal biopsies is a strength in that this is the population from which cases are most likely to arise. Additionally, we have confidence in the validity of our exposure and outcome measurements as the pathology results were derived through consistent, well-defined protocols across samples, and the PM metrics are from federal resources involving numerous quality checks.

In conclusion, we found that exposure to ambient PM_2.5_ and PM_10_ concentrations is positively if moderately associated with EoE case status in study of a large, national pathology database. The association could be a direct effect of particulate matter, could be an indirect effect either through causation or increase diagnosis, and the associations include the null value after adjustment. A large effect of particulate air pollution to cause the epidemic increase in EoE that has occurred in recent decades is not well supported by these data. However, further investigation of the potential role of specific components of air pollution as well as additional sources of environmental exposures, such as water, processed foods, etc. is warranted, particularly if longitudinal data are available. Our results and methods can serve as a tool to continue investigations into environmental underpinnings of EoE etiology.

## Data Availability

The datasets presented in this article are not readily available because the data are not publicly available due to restrictions from the data owner. Requests to access the datasets should be directed to evan_dellon@med.unc.edu.

## Supplementary material

Supplement Figure 1 Choropleth map of the estimated mean PM2.5 levels by year and contiguous United States county in tons emitted per year.

Supplemental Figure 2 Choropleth map of the estimated mean PM10 levels by year and contiguous United States county in tons emitted per year.
