# Supplementary figures and images for: Particulate matter as a possible risk factor for eosinophilic esophagitis
Source: Front Allergy. 2025 Sep 18;6:1675928. doi: 10.3389/falgy.2025.1675928 (PMC12488640; doi:10.3389/falgy.2025.1675928)

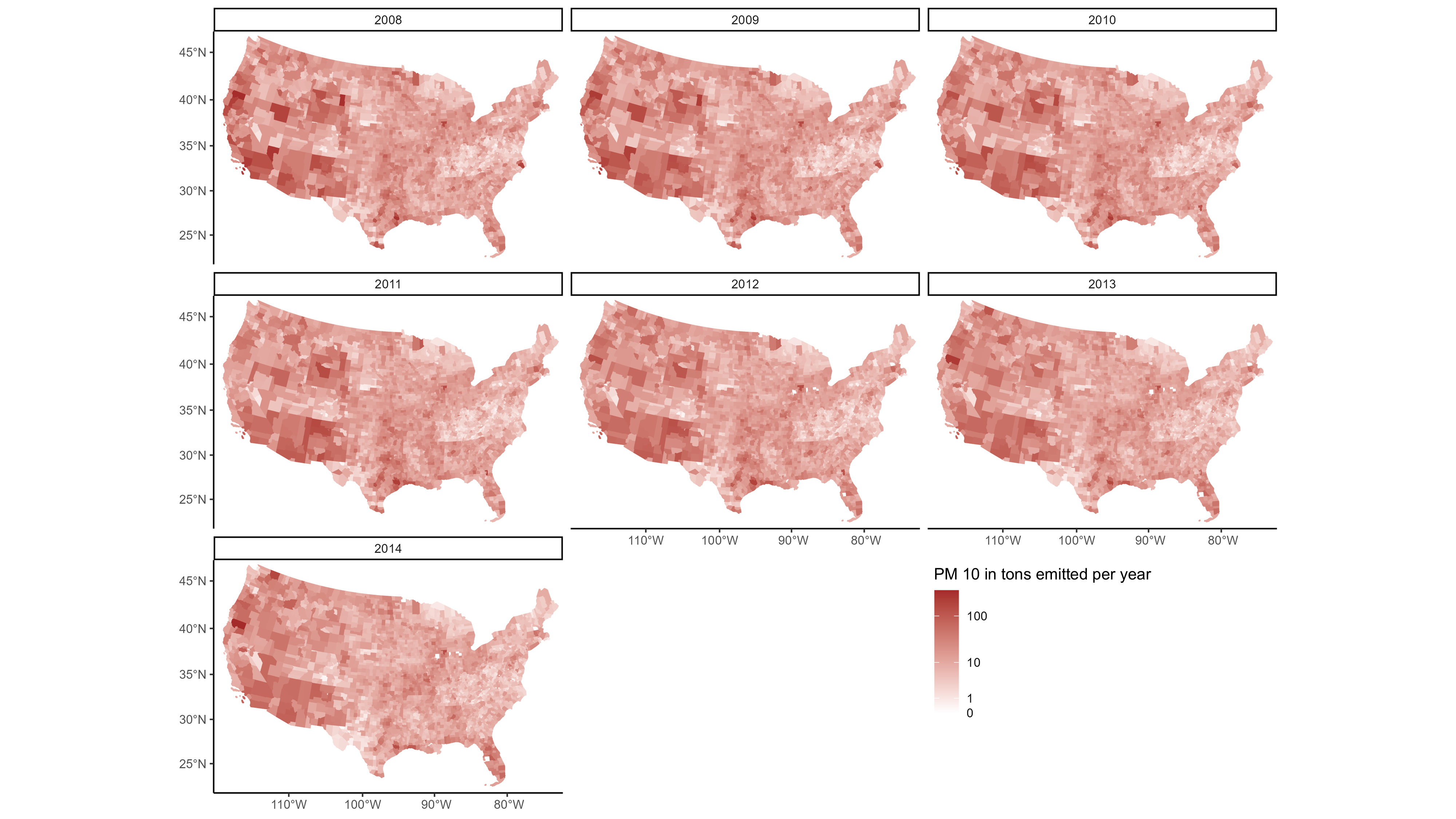

Supplement: Supplement Figure 1 — Choropleth map of the estimated mean PM2.5 levels by year and contiguous United States county in tons emitted per year. [file Image1.tif]

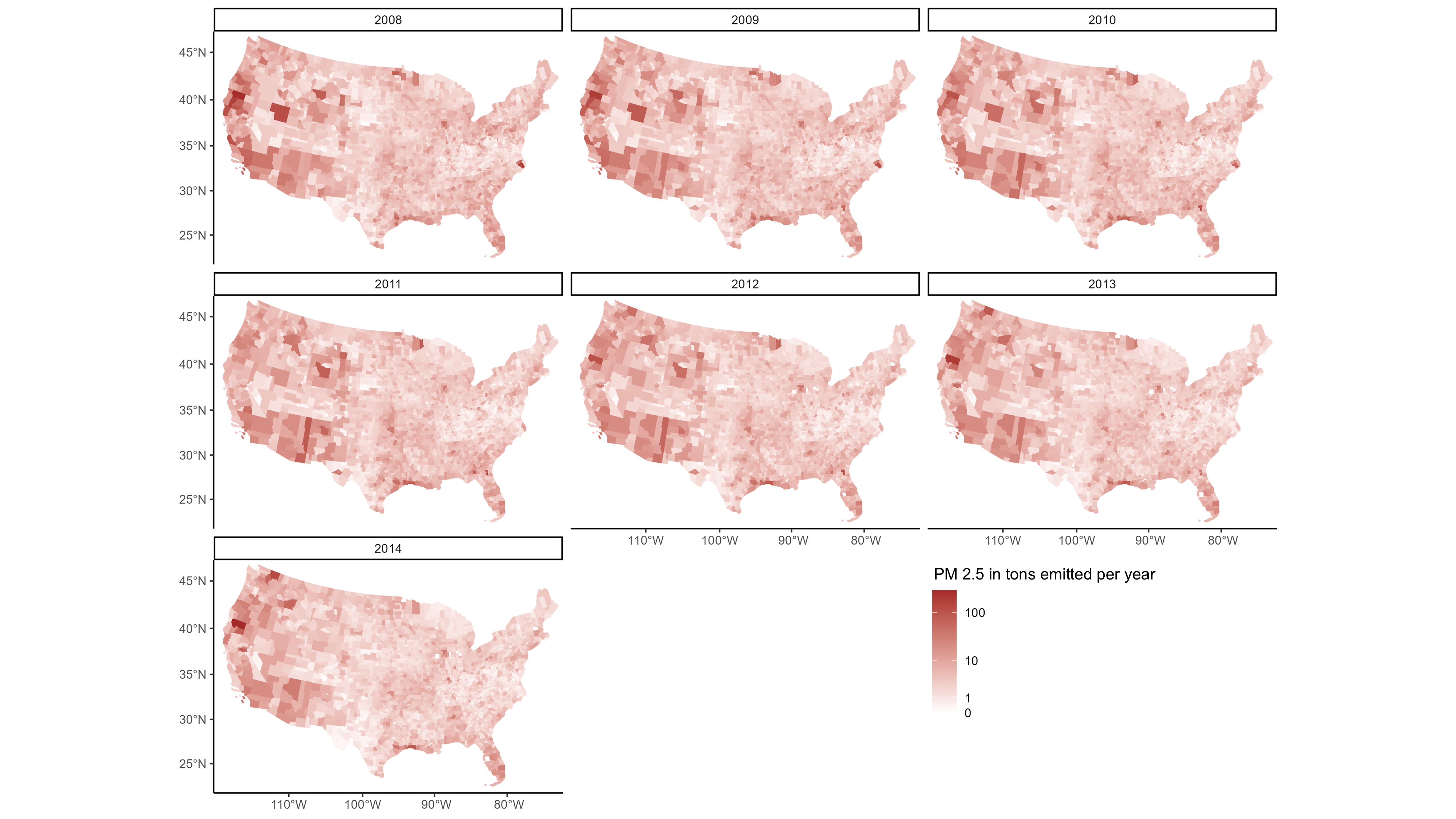

Supplement: Supplemental Figure 2 — Choropleth map of the estimated mean PM10 levels by year and contiguous United States county in tons emitted per year. [file Image2.tif]
